# Ethyl 8′′-chloro-1′-methyl-2,12′′-dioxo-12′′*H*-di­spiro­[indoline-3,2′-pyrrolidine- 3′,6′′-indolo[2,1-*b*]quinazoline]-4′-carboxyl­ate

**DOI:** 10.1107/S1600536813015146

**Published:** 2013-06-08

**Authors:** Piskala Subburaman Kannan, Srinu Lanka, Sathiah Thennarasu, E. Govindan, Arunachalathevar SubbiahPandi

**Affiliations:** a Department of Physics, S.M.K. Fomra Institute of Technology, Thaiyur, Chennai 603 103, India; b Organic Chemistry Division, CSIR Central Leather Research Institute, Adyar, Chennai 600 020, India; c Department of Physics, Presidency College (Autonomous), Chennai 600 005, India

## Abstract

In the title compound, C_29_H_23_ClN_4_O_4_, the quinazoline-indole system and the indolin-2-one system are each essentially planar, with maximum deviations from their mean planes of 0.150 (2) and 0.072 (2) Å, respectively. The central pyrrolidine ring adopts a twisted conformation on the C—C bond involving the spiro C atoms. Its mean plane forms dihedral angles of 83.37 (9) and 86.56 (8)°, respectively, with the indole rings of the indolin-2-one and quinazoline-indole systems. In the crystal, mol­ecules are linked *via* pairs of N—H⋯O hydrogen bonds, forming inversion dimers. The dimers are linked *via* C—H⋯O hydrogen bonds, forming chains propagating along [001].

## Related literature

For quinazoline structures, see: Li & Feng (2009 ▶); Li *et al.* (2010 ▶); Priya *et al.* (2011*a* ▶). For the biological activity of quinazoline derivatives, see: Wolfe *et al.* (1990 ▶); Tereshima *et al.* (1995 ▶); Pandeya *et al.* (1999 ▶); Priya *et al.* (2011*b* ▶). For ring conformations, see: Cremer & Pople (1975 ▶).
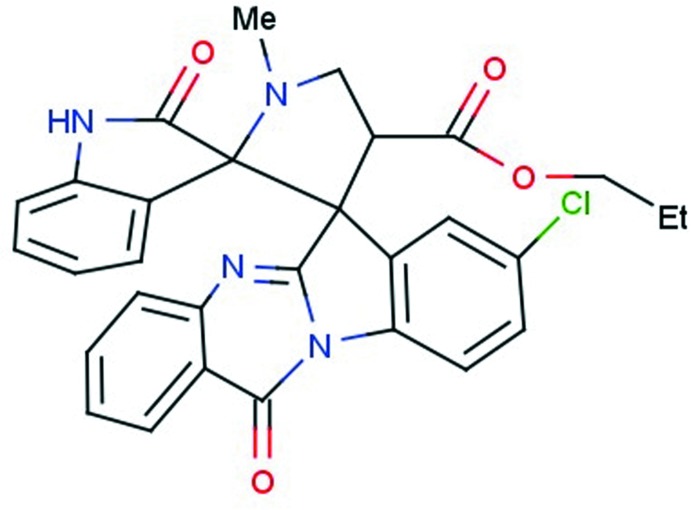


## Experimental

### 

#### Crystal data

C_29_H_23_ClN_4_O_4_
*M* *_r_* = 526.96
Triclinic, 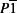

*a* = 8.9341 (9) Å
*b* = 11.7697 (12) Å
*c* = 13.3828 (14) Å
α = 72.776 (5)°
β = 89.574 (5)°
γ = 74.995 (5)°
*V* = 1294.6 (2) Å^3^
*Z* = 2
Mo *K*α radiation
μ = 0.19 mm^−1^
*T* = 293 K
0.30 × 0.25 × 0.20 mm

#### Data collection

Bruker SMART APEXII area-detector diffractometer
Absorption correction: multi-scan (*SADABS*; Bruker, 2008 ▶) *T*_min_ = 0.945, *T*_max_ = 0.963
16756 measured reflections
6722 independent reflections
5191 reflections with *I* > 2σ(*I*)
*R*_int_ = 0.028

#### Refinement

*R*[*F* ^2^ > 2σ(*F* ^2^)] = 0.053
*wR*(*F* ^2^) = 0.161
*S* = 1.04
6722 reflections
346 parameters
H-atom parameters constrained
Δρ_max_ = 0.59 e Å^−3^
Δρ_min_ = −0.50 e Å^−3^

### 

Data collection: *APEX2* (Bruker, 2008 ▶); cell refinement: *SAINT* (Bruker, 2008 ▶); data reduction: *SAINT*; program(s) used to solve structure: *SHELXS97* (Sheldrick, 2008 ▶); program(s) used to refine structure: *SHELXL97* (Sheldrick, 2008 ▶); molecular graphics: *ORTEP-3 for Windows* (Farrugia, 2012 ▶) and *PLATON* (Spek, 2009 ▶); software used to prepare material for publication: *SHELXL97* and *PLATON*.

## Supplementary Material

Crystal structure: contains datablock(s) global, I. DOI: 10.1107/S1600536813015146/su2600sup1.cif

Structure factors: contains datablock(s) I. DOI: 10.1107/S1600536813015146/su2600Isup2.hkl

Additional supplementary materials:  crystallographic information; 3D view; checkCIF report

## Figures and Tables

**Table 1:** Hydrogen-bond geometry (Å, °)

*D*—H⋯*A*	*D*—H	H⋯*A*	*D*⋯*A*	*D*—H⋯*A*
N4—H4⋯O4^i^	0.86	1.98	2.808 (2)	160
C20—H20*C*⋯O1^ii^	0.96	2.53	3.369 (6)	146

Symmetry codes: (i) 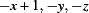; (ii) 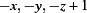.

**Fig. 1.**
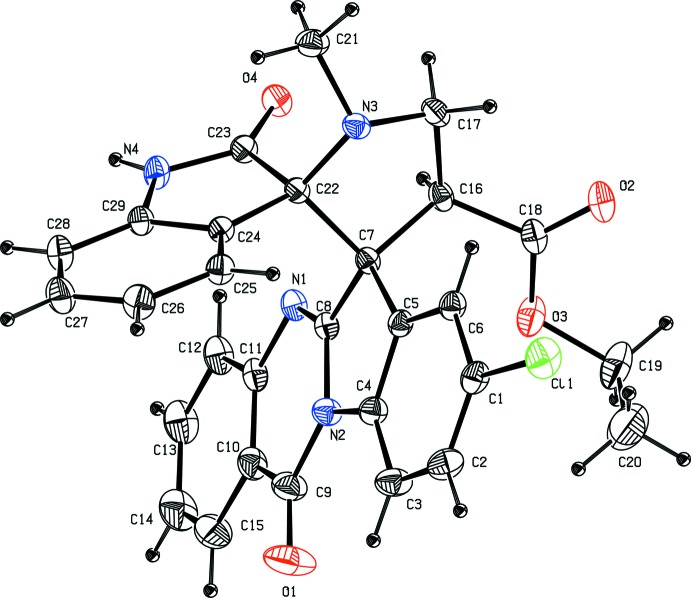
The molecular structure of the title molecule, with the atom labelling. Displacement ellipsoids are drawn at the 30% probability level.

**Fig. 2.**
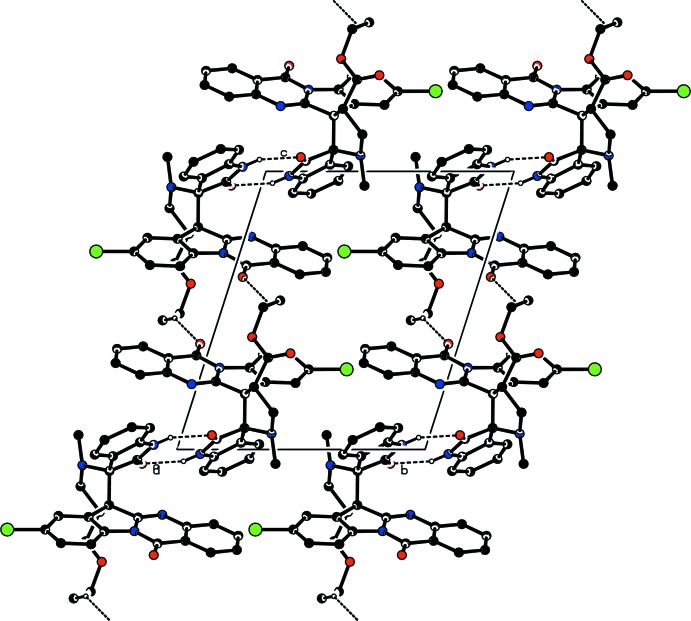
A view along the a axis of the crystal packing of the title compound, showing the N-H···O and C-H···O hydrogen bonds as dashed lines.

## supplementary crystallographic information

### Comment

Quinazolines are an important class of fused heterocycles with a wide range of biological activities such as anticancer (Wolfe *et al.*, 1990), anti-inflammatory (Tereshima *et al.*, 1995), and anti-HIV (Pandeya *et al.*, 1999). In addition, quinazolines exhibit antibacterial and anti-fungal activities (Priya *et al.*, 2011*b*).

In view of their importance and in continuation of our work on the crystal structure analysis of pyrrolidine and quinazoline derivatives, we report herein on the crystal structure of the title compound.

The molecular structure of the title molecule is illustrated in Fig. 1. The quinazoline-indole systems (N1/N2/C1-C15) and indolin-2-one system (N4/C22-C29), are essentially planar, with maximum deviations of 0.150 (2) Å for atom C1 and 0.072 (2) Å for atom C22 in the respective systems.

The central pyrrolidine ring (N3/C7/C16/C17/C22) is twisted on bond C7-C22 with puckering parameters of q_2_ = 0.4458 (2)Å, φ = 314.2 (2)° (Cremer & Pople, 1975). The mean plane of this ring forms dihedral angles of 83.37 (9) and 86.56 (8)° with the two indole rings (N4/C22-C29) and (N2/C1-C8), respectively. This clearly shows that the central pyrrolidine ring system and the two indole rings are almost perpendicular to one another.

In the crystal, molecules are linked via pairs of N-H···O hydrogen bonds forming inversion dimers. The dimers are linked via C-H···O hydrogen bonds forming chains propagating along the c axis direction. (Table 1).

### Experimental

Isatin (0.25 mmol) , sarcosine (0.3 mmol), (E)-ethyl 2-(8-chloro-12-oxoindolo[2,1-b]quinazolin-6(12H)-ylidene)acetate (0.25 mmol) in ethanol were refluxed for 120 min. The progress of the reaction was followed by TLC. After completion, the solvent was removed under reduced pressure and the resulting crude product was subjected to column chromatography eluted with n-hexane/EtOAc (8.5:1.5). The product was recrystallised from ethanol. Single crystals suitable for X-ray diffraction were obtained by slow evaporation of the solution of the title compound in ethanol at room temperature.

### Refinement

All the H atoms were fixed geometrically and allowed to ride on their parent C atoms: N-H = 0.86 Å, C—H = 0.93–0.97 Å with *U*_iso_(H) = 1.5*U*_eq_(C-methyl) and = 1.2*U*_eq_(N,C) for other H atoms. The positions of the methyl hydrogens were optimized rotationally.

### Crystal data

**Table:**

C_29_H_23_ClN_4_O_4_	*Z* = 2
*M**_r_* = 526.96	*F*(000) = 548
Triclinic, *P*1	*D*_x_ = 1.352 Mg m^−^^3^
Hall symbol: -P 1	Mo *K*α radiation, λ = 0.71073 Å
*a* = 8.9341 (9) Å	Cell parameters from 6722 reflections
*b* = 11.7697 (12) Å	θ = 1.6–29.7°
*c* = 13.3828 (14) Å	µ = 0.19 mm^−^^1^
α = 72.776 (5)°	*T* = 293 K
β = 89.574 (5)°	Block, colourless
γ = 74.995 (5)°	0.30 × 0.25 × 0.20 mm
*V* = 1294.6 (2) Å^3^	

### Data collection

**Table:**

Bruker SMART APEXII area-detector diffractometer	6722 independent reflections
Radiation source: fine-focus sealed tube	5191 reflections with *I* > 2σ(*I*)
Graphite monochromator	*R*_int_ = 0.028
ω and φ scans	θ_max_ = 29.7°, θ_min_ = 1.6°
Absorption correction: multi-scan (*SADABS*; Bruker, 2008)	*h* = −12→12
*T*_min_ = 0.945, *T*_max_ = 0.963	*k* = −16→16
16756 measured reflections	*l* = −18→18

### Refinement

**Table:**

Refinement on *F*^2^	Secondary atom site location: difference Fourier map
Least-squares matrix: full	Hydrogen site location: inferred from neighbouring sites
*R*[*F*^2^ > 2σ(*F*^2^)] = 0.053	H-atom parameters constrained
*wR*(*F*^2^) = 0.161	*w* = 1/[σ^2^(*F*_o_^2^) + (0.0796*P*)^2^ + 0.4319*P*] where *P* = (*F*_o_^2^ + 2*F*_c_^2^)/3
*S* = 1.04	(Δ/σ)_max_ < 0.001
6722 reflections	Δρ_max_ = 0.59 e Å^−^^3^
346 parameters	Δρ_min_ = −0.50 e Å^−^^3^
0 restraints	Extinction correction: *SHELXL*, Fc^*^=kFc[1+0.001xFc^2^λ^3^/sin(2θ)]^-1/4^
Primary atom site location: structure-invariant direct methods	Extinction coefficient: 0.022 (3)

### Special details

**Table:**

Geometry. All esds (except the esd in the dihedral angle between two l.s. planes) are estimated using the full covariance matrix. The cell esds are taken into account individually in the estimation of esds in distances, angles and torsion angles; correlations between esds in cell parameters are only used when they are defined by crystal symmetry. An approximate (isotropic) treatment of cell esds is used for estimating esds involving l.s. planes.
Refinement. Refinement of F^2^ against ALL reflections. The weighted R-factor wR and goodness of fit S are based on F^2^, conventional R-factors R are based on F, with F set to zero for negative F^2^. The threshold expression of F^2^ > 2sigma(F^2^) is used only for calculating R-factors(gt) etc. and is not relevant to the choice of reflections for refinement. R-factors based on F^2^ are statistically about twice as large as those based on F, and R- factors based on ALL data will be even larger.

### Fractional atomic coordinates and isotropic or equivalent isotropic displacement parameters (Å^2^)

**Table:**

	*x*	*y*	*z*	*U*_iso_*/*U*_eq_	
C1	−0.0433 (2)	0.42790 (17)	0.28795 (14)	0.0474 (4)	
C2	−0.1275 (2)	0.3468 (2)	0.33683 (17)	0.0592 (5)	
H2	−0.2181	0.3749	0.3671	0.071*	
C3	−0.0777 (2)	0.2237 (2)	0.34100 (18)	0.0587 (5)	
H3	−0.1331	0.1679	0.3740	0.070*	
C4	0.0570 (2)	0.18699 (16)	0.29445 (13)	0.0429 (4)	
C5	0.13927 (18)	0.26916 (15)	0.24246 (12)	0.0364 (3)	
C6	0.08974 (19)	0.39168 (15)	0.23894 (13)	0.0411 (4)	
H6	0.1439	0.4479	0.2048	0.049*	
C7	0.28025 (17)	0.20187 (13)	0.19999 (12)	0.0340 (3)	
C8	0.27475 (18)	0.06884 (14)	0.24454 (12)	0.0361 (3)	
C9	0.0961 (3)	−0.04311 (19)	0.33813 (18)	0.0591 (5)	
C10	0.2124 (3)	−0.15153 (17)	0.32853 (15)	0.0529 (5)	
C11	0.3470 (2)	−0.13953 (15)	0.27742 (13)	0.0445 (4)	
C12	0.4550 (3)	−0.24456 (18)	0.26869 (17)	0.0568 (5)	
H12	0.5443	−0.2371	0.2341	0.068*	
C13	0.4304 (3)	−0.3585 (2)	0.3106 (2)	0.0713 (7)	
H13	0.5035	−0.4283	0.3049	0.086*	
C14	0.2976 (4)	−0.3709 (2)	0.3616 (2)	0.0799 (8)	
H14	0.2822	−0.4490	0.3899	0.096*	
C15	0.1884 (3)	−0.2690 (2)	0.3706 (2)	0.0730 (7)	
H15	0.0989	−0.2779	0.4044	0.088*	
C16	0.43389 (18)	0.22977 (15)	0.22466 (14)	0.0402 (3)	
H16	0.5202	0.1596	0.2226	0.048*	
C17	0.4423 (2)	0.34102 (17)	0.13268 (15)	0.0472 (4)	
H17A	0.4313	0.4133	0.1558	0.057*	
H17B	0.5408	0.3245	0.1015	0.057*	
C18	0.4471 (2)	0.24906 (17)	0.32997 (15)	0.0485 (4)	
C19	0.4183 (4)	0.1643 (3)	0.51159 (19)	0.0871 (9)	
H19A	0.4540	0.0815	0.5601	0.104*	
H19B	0.4909	0.2102	0.5196	0.104*	
C20	0.2652 (5)	0.2240 (6)	0.5358 (3)	0.147 (2)	
H20A	0.2310	0.3065	0.4886	0.220*	
H20B	0.2695	0.2268	0.6067	0.220*	
H20C	0.1936	0.1782	0.5281	0.220*	
C21	0.3407 (2)	0.41556 (18)	−0.05185 (15)	0.0536 (5)	
H21A	0.4363	0.3673	−0.0684	0.080*	
H21B	0.3474	0.4983	−0.0619	0.080*	
H21C	0.2566	0.4173	−0.0970	0.080*	
C22	0.27177 (17)	0.24447 (13)	0.07722 (12)	0.0336 (3)	
C23	0.38921 (18)	0.14670 (14)	0.03777 (13)	0.0387 (3)	
C24	0.12036 (17)	0.24951 (14)	0.02419 (12)	0.0353 (3)	
C25	−0.02790 (19)	0.32460 (17)	0.01850 (14)	0.0446 (4)	
H25	−0.0480	0.3877	0.0493	0.054*	
C26	−0.1467 (2)	0.3041 (2)	−0.03426 (17)	0.0563 (5)	
H26	−0.2474	0.3541	−0.0387	0.068*	
C27	−0.1176 (2)	0.2107 (2)	−0.08016 (17)	0.0598 (5)	
H27	−0.1995	0.1973	−0.1134	0.072*	
C28	0.0317 (2)	0.13666 (18)	−0.07758 (16)	0.0526 (4)	
H28	0.0520	0.0748	−0.1097	0.063*	
C29	0.14840 (19)	0.15821 (15)	−0.02564 (13)	0.0397 (3)	
N1	0.37705 (16)	−0.02506 (12)	0.23485 (11)	0.0415 (3)	
N2	0.13797 (17)	0.06638 (13)	0.29362 (12)	0.0436 (3)	
N3	0.31307 (16)	0.36062 (12)	0.05689 (11)	0.0391 (3)	
N4	0.30806 (16)	0.10001 (13)	−0.01648 (12)	0.0442 (3)	
H4	0.3491	0.0415	−0.0425	0.053*	
O1	−0.0250 (3)	−0.04254 (17)	0.37966 (19)	0.0995 (7)	
O2	0.4841 (2)	0.33332 (18)	0.34553 (14)	0.0815 (5)	
O3	0.4134 (2)	0.15839 (14)	0.40408 (11)	0.0665 (4)	
O4	0.53071 (13)	0.12214 (12)	0.05120 (11)	0.0489 (3)	
Cl1	−0.10529 (7)	0.58059 (5)	0.28817 (5)	0.0708 (2)	

### Atomic displacement parameters (Å^2^)

**Table:**

	*U*^11^	*U*^22^	*U*^33^	*U*^12^	*U*^13^	*U*^23^
C1	0.0454 (9)	0.0461 (10)	0.0500 (9)	−0.0007 (7)	0.0045 (7)	−0.0236 (8)
C2	0.0483 (10)	0.0634 (13)	0.0642 (12)	−0.0046 (9)	0.0200 (9)	−0.0265 (10)
C3	0.0527 (11)	0.0557 (11)	0.0689 (12)	−0.0159 (9)	0.0245 (9)	−0.0201 (9)
C4	0.0429 (9)	0.0403 (9)	0.0457 (9)	−0.0095 (7)	0.0066 (7)	−0.0149 (7)
C5	0.0353 (7)	0.0385 (8)	0.0375 (7)	−0.0075 (6)	0.0037 (6)	−0.0165 (6)
C6	0.0409 (8)	0.0396 (8)	0.0444 (8)	−0.0061 (7)	0.0052 (6)	−0.0192 (7)
C7	0.0311 (7)	0.0305 (7)	0.0425 (8)	−0.0064 (6)	0.0032 (6)	−0.0159 (6)
C8	0.0373 (7)	0.0334 (8)	0.0384 (7)	−0.0089 (6)	−0.0011 (6)	−0.0126 (6)
C9	0.0665 (12)	0.0476 (11)	0.0668 (13)	−0.0257 (9)	0.0176 (10)	−0.0143 (9)
C10	0.0693 (12)	0.0378 (9)	0.0529 (10)	−0.0189 (8)	−0.0002 (9)	−0.0120 (7)
C11	0.0527 (10)	0.0350 (8)	0.0450 (9)	−0.0100 (7)	−0.0102 (7)	−0.0124 (7)
C12	0.0604 (11)	0.0400 (10)	0.0674 (12)	−0.0052 (8)	−0.0116 (9)	−0.0190 (8)
C13	0.0839 (16)	0.0370 (10)	0.0862 (16)	−0.0048 (10)	−0.0139 (13)	−0.0183 (10)
C14	0.111 (2)	0.0353 (11)	0.0895 (17)	−0.0228 (12)	−0.0025 (15)	−0.0101 (10)
C15	0.0948 (18)	0.0485 (12)	0.0788 (15)	−0.0326 (12)	0.0131 (13)	−0.0128 (10)
C16	0.0326 (7)	0.0389 (8)	0.0545 (9)	−0.0075 (6)	0.0012 (6)	−0.0238 (7)
C17	0.0425 (9)	0.0467 (9)	0.0632 (11)	−0.0206 (7)	0.0100 (8)	−0.0253 (8)
C18	0.0416 (9)	0.0480 (10)	0.0598 (11)	−0.0068 (7)	−0.0062 (7)	−0.0264 (8)
C19	0.121 (2)	0.0858 (18)	0.0492 (12)	−0.0113 (16)	−0.0141 (13)	−0.0274 (12)
C20	0.112 (3)	0.275 (6)	0.101 (3)	−0.081 (4)	0.033 (2)	−0.102 (3)
C21	0.0618 (11)	0.0452 (10)	0.0564 (11)	−0.0203 (9)	0.0184 (9)	−0.0143 (8)
C22	0.0305 (7)	0.0305 (7)	0.0419 (8)	−0.0063 (5)	0.0063 (5)	−0.0160 (6)
C23	0.0360 (8)	0.0351 (8)	0.0468 (8)	−0.0059 (6)	0.0074 (6)	−0.0186 (6)
C24	0.0336 (7)	0.0335 (7)	0.0383 (7)	−0.0068 (6)	0.0029 (6)	−0.0122 (6)
C25	0.0372 (8)	0.0463 (9)	0.0495 (9)	−0.0040 (7)	0.0027 (7)	−0.0197 (7)
C26	0.0345 (8)	0.0670 (13)	0.0640 (12)	−0.0025 (8)	−0.0044 (8)	−0.0244 (10)
C27	0.0472 (10)	0.0687 (13)	0.0668 (12)	−0.0156 (9)	−0.0106 (9)	−0.0255 (10)
C28	0.0538 (10)	0.0503 (10)	0.0594 (11)	−0.0121 (8)	−0.0059 (8)	−0.0268 (8)
C29	0.0401 (8)	0.0360 (8)	0.0429 (8)	−0.0072 (6)	0.0003 (6)	−0.0144 (6)
N1	0.0417 (7)	0.0338 (7)	0.0487 (8)	−0.0065 (6)	−0.0035 (6)	−0.0152 (6)
N2	0.0445 (8)	0.0369 (7)	0.0501 (8)	−0.0116 (6)	0.0087 (6)	−0.0139 (6)
N3	0.0389 (7)	0.0335 (7)	0.0485 (7)	−0.0120 (5)	0.0104 (5)	−0.0161 (5)
N4	0.0410 (7)	0.0406 (8)	0.0554 (8)	−0.0021 (6)	0.0024 (6)	−0.0287 (6)
O1	0.0973 (14)	0.0625 (11)	0.1478 (19)	−0.0391 (10)	0.0673 (13)	−0.0317 (11)
O2	0.1052 (14)	0.0883 (12)	0.0827 (11)	−0.0511 (11)	0.0076 (10)	−0.0514 (10)
O3	0.0946 (12)	0.0566 (9)	0.0479 (8)	−0.0149 (8)	−0.0109 (7)	−0.0195 (6)
O4	0.0335 (6)	0.0490 (7)	0.0715 (8)	−0.0064 (5)	0.0110 (5)	−0.0337 (6)
Cl1	0.0664 (3)	0.0550 (3)	0.0940 (4)	0.0023 (2)	0.0119 (3)	−0.0436 (3)

### Geometric parameters (Å, º)

**Table:**

C1—C2	1.378 (3)	C17—N3	1.468 (2)
C1—C6	1.384 (2)	C17—H17A	0.9700
C1—Cl1	1.7390 (19)	C17—H17B	0.9700
C2—C3	1.385 (3)	C18—O2	1.195 (2)
C2—H2	0.9300	C18—O3	1.322 (2)
C3—C4	1.379 (3)	C19—C20	1.449 (5)
C3—H3	0.9300	C19—O3	1.462 (3)
C4—C5	1.386 (2)	C19—H19A	0.9700
C4—N2	1.420 (2)	C19—H19B	0.9700
C5—C6	1.381 (2)	C20—H20A	0.9600
C5—C7	1.509 (2)	C20—H20B	0.9600
C6—H6	0.9300	C20—H20C	0.9600
C7—C8	1.515 (2)	C21—N3	1.455 (2)
C7—C16	1.551 (2)	C21—H21A	0.9600
C7—C22	1.566 (2)	C21—H21B	0.9600
C8—N1	1.278 (2)	C21—H21C	0.9600
C8—N2	1.386 (2)	C22—N3	1.4555 (19)
C9—O1	1.212 (3)	C22—C24	1.512 (2)
C9—N2	1.395 (2)	C22—C23	1.559 (2)
C9—C10	1.460 (3)	C23—O4	1.2237 (19)
C10—C11	1.398 (3)	C23—N4	1.344 (2)
C10—C15	1.402 (3)	C24—C25	1.377 (2)
C11—C12	1.392 (3)	C24—C29	1.395 (2)
C11—N1	1.394 (2)	C25—C26	1.388 (3)
C12—C13	1.367 (3)	C25—H25	0.9300
C12—H12	0.9300	C26—C27	1.379 (3)
C13—C14	1.383 (4)	C26—H26	0.9300
C13—H13	0.9300	C27—C28	1.386 (3)
C14—C15	1.372 (4)	C27—H27	0.9300
C14—H14	0.9300	C28—C29	1.374 (2)
C15—H15	0.9300	C28—H28	0.9300
C16—C18	1.503 (2)	C29—N4	1.402 (2)
C16—C17	1.527 (2)	N4—H4	0.8600
C16—H16	0.9800		
			
C2—C1—C6	122.13 (17)	O2—C18—O3	124.37 (19)
C2—C1—Cl1	119.09 (14)	O2—C18—C16	125.3 (2)
C6—C1—Cl1	118.78 (15)	O3—C18—C16	110.28 (15)
C1—C2—C3	120.27 (17)	C20—C19—O3	110.2 (2)
C1—C2—H2	119.9	C20—C19—H19A	109.6
C3—C2—H2	119.9	O3—C19—H19A	109.6
C4—C3—C2	117.58 (18)	C20—C19—H19B	109.6
C4—C3—H3	121.2	O3—C19—H19B	109.6
C2—C3—H3	121.2	H19A—C19—H19B	108.1
C3—C4—C5	122.23 (17)	C19—C20—H20A	109.5
C3—C4—N2	128.84 (17)	C19—C20—H20B	109.5
C5—C4—N2	108.87 (14)	H20A—C20—H20B	109.5
C6—C5—C4	120.00 (15)	C19—C20—H20C	109.5
C6—C5—C7	130.09 (14)	H20A—C20—H20C	109.5
C4—C5—C7	109.87 (14)	H20B—C20—H20C	109.5
C5—C6—C1	117.73 (16)	N3—C21—H21A	109.5
C5—C6—H6	121.1	N3—C21—H21B	109.5
C1—C6—H6	121.1	H21A—C21—H21B	109.5
C5—C7—C8	101.82 (12)	N3—C21—H21C	109.5
C5—C7—C16	114.53 (12)	H21A—C21—H21C	109.5
C8—C7—C16	116.11 (12)	H21B—C21—H21C	109.5
C5—C7—C22	112.56 (12)	N3—C22—C24	116.72 (13)
C8—C7—C22	111.93 (12)	N3—C22—C23	113.52 (12)
C16—C7—C22	100.39 (12)	C24—C22—C23	101.29 (12)
N1—C8—N2	125.77 (15)	N3—C22—C7	100.51 (11)
N1—C8—C7	125.36 (14)	C24—C22—C7	115.11 (12)
N2—C8—C7	108.82 (13)	C23—C22—C7	110.10 (12)
O1—C9—N2	121.0 (2)	O4—C23—N4	126.97 (15)
O1—C9—C10	126.37 (19)	O4—C23—C22	124.83 (14)
N2—C9—C10	112.66 (17)	N4—C23—C22	108.14 (13)
C11—C10—C15	119.5 (2)	C25—C24—C29	119.60 (15)
C11—C10—C9	120.78 (16)	C25—C24—C22	131.85 (15)
C15—C10—C9	119.7 (2)	C29—C24—C22	108.54 (13)
C12—C11—N1	118.71 (18)	C24—C25—C26	118.58 (17)
C12—C11—C10	119.41 (17)	C24—C25—H25	120.7
N1—C11—C10	121.88 (16)	C26—C25—H25	120.7
C13—C12—C11	120.3 (2)	C27—C26—C25	120.98 (17)
C13—C12—H12	119.8	C27—C26—H26	119.5
C11—C12—H12	119.8	C25—C26—H26	119.5
C12—C13—C14	120.5 (2)	C26—C27—C28	121.07 (17)
C12—C13—H13	119.8	C26—C27—H27	119.5
C14—C13—H13	119.8	C28—C27—H27	119.5
C15—C14—C13	120.5 (2)	C29—C28—C27	117.41 (18)
C15—C14—H14	119.7	C29—C28—H28	121.3
C13—C14—H14	119.7	C27—C28—H28	121.3
C14—C15—C10	119.7 (2)	C28—C29—C24	122.30 (16)
C14—C15—H15	120.2	C28—C29—N4	127.95 (16)
C10—C15—H15	120.2	C24—C29—N4	109.72 (14)
C18—C16—C17	113.67 (14)	C8—N1—C11	116.60 (15)
C18—C16—C7	114.51 (14)	C8—N2—C9	122.30 (15)
C17—C16—C7	104.53 (13)	C8—N2—C4	110.19 (13)
C18—C16—H16	107.9	C9—N2—C4	127.47 (16)
C17—C16—H16	107.9	C21—N3—C22	114.39 (13)
C7—C16—H16	107.9	C21—N3—C17	114.30 (14)
N3—C17—C16	105.79 (12)	C22—N3—C17	107.73 (13)
N3—C17—H17A	110.6	C23—N4—C29	111.88 (13)
C16—C17—H17A	110.6	C23—N4—H4	124.1
N3—C17—H17B	110.6	C29—N4—H4	124.1
C16—C17—H17B	110.6	C18—O3—C19	117.25 (19)
H17A—C17—H17B	108.7		
			
C6—C1—C2—C3	2.0 (3)	C5—C7—C22—C23	−162.07 (12)
Cl1—C1—C2—C3	−177.74 (17)	C8—C7—C22—C23	−48.09 (16)
C1—C2—C3—C4	−0.3 (3)	C16—C7—C22—C23	75.70 (14)
C2—C3—C4—C5	−1.7 (3)	N3—C22—C23—O4	45.3 (2)
C2—C3—C4—N2	175.36 (19)	C24—C22—C23—O4	171.30 (16)
C3—C4—C5—C6	2.1 (3)	C7—C22—C23—O4	−66.4 (2)
N2—C4—C5—C6	−175.50 (14)	N3—C22—C23—N4	−131.88 (14)
C3—C4—C5—C7	−179.89 (17)	C24—C22—C23—N4	−5.93 (16)
N2—C4—C5—C7	2.50 (19)	C7—C22—C23—N4	116.34 (14)
C4—C5—C6—C1	−0.4 (2)	N3—C22—C24—C25	−50.0 (2)
C7—C5—C6—C1	−177.96 (16)	C23—C22—C24—C25	−173.76 (17)
C2—C1—C6—C5	−1.6 (3)	C7—C22—C24—C25	67.5 (2)
Cl1—C1—C6—C5	178.12 (12)	N3—C22—C24—C29	129.99 (14)
C6—C5—C7—C8	172.28 (16)	C23—C22—C24—C29	6.19 (16)
C4—C5—C7—C8	−5.45 (16)	C7—C22—C24—C29	−112.53 (15)
C6—C5—C7—C16	46.1 (2)	C29—C24—C25—C26	2.1 (3)
C4—C5—C7—C16	−131.60 (15)	C22—C24—C25—C26	−177.94 (17)
C6—C5—C7—C22	−67.7 (2)	C24—C25—C26—C27	0.0 (3)
C4—C5—C7—C22	114.56 (15)	C25—C26—C27—C28	−1.8 (4)
C5—C7—C8—N1	−175.82 (15)	C26—C27—C28—C29	1.4 (3)
C16—C7—C8—N1	−50.7 (2)	C27—C28—C29—C24	0.8 (3)
C22—C7—C8—N1	63.73 (19)	C27—C28—C29—N4	−176.79 (19)
C5—C7—C8—N2	6.56 (16)	C25—C24—C29—C28	−2.5 (3)
C16—C7—C8—N2	131.66 (14)	C22—C24—C29—C28	177.49 (16)
C22—C7—C8—N2	−113.89 (14)	C25—C24—C29—N4	175.40 (15)
O1—C9—C10—C11	179.2 (2)	C22—C24—C29—N4	−4.56 (19)
N2—C9—C10—C11	−1.1 (3)	N2—C8—N1—C11	0.2 (2)
O1—C9—C10—C15	−0.5 (4)	C7—C8—N1—C11	−177.04 (14)
N2—C9—C10—C15	179.20 (19)	C12—C11—N1—C8	179.91 (15)
C15—C10—C11—C12	0.3 (3)	C10—C11—N1—C8	−0.2 (2)
C9—C10—C11—C12	−179.39 (18)	N1—C8—N2—C9	−0.7 (3)
C15—C10—C11—N1	−179.57 (18)	C7—C8—N2—C9	176.92 (16)
C9—C10—C11—N1	0.8 (3)	N1—C8—N2—C4	176.85 (15)
N1—C11—C12—C13	179.18 (18)	C7—C8—N2—C4	−5.54 (18)
C10—C11—C12—C13	−0.7 (3)	O1—C9—N2—C8	−179.2 (2)
C11—C12—C13—C14	0.5 (4)	C10—C9—N2—C8	1.1 (3)
C12—C13—C14—C15	0.1 (4)	O1—C9—N2—C4	3.7 (4)
C13—C14—C15—C10	−0.5 (4)	C10—C9—N2—C4	−176.00 (17)
C11—C10—C15—C14	0.3 (4)	C3—C4—N2—C8	−175.45 (19)
C9—C10—C15—C14	180.0 (2)	C5—C4—N2—C8	1.95 (19)
C5—C7—C16—C18	35.44 (19)	C3—C4—N2—C9	1.9 (3)
C8—C7—C16—C18	−82.88 (17)	C5—C4—N2—C9	179.32 (18)
C22—C7—C16—C18	156.27 (14)	C24—C22—N3—C21	−64.33 (18)
C5—C7—C16—C17	−89.58 (16)	C23—C22—N3—C21	52.94 (19)
C8—C7—C16—C17	152.10 (14)	C7—C22—N3—C21	170.46 (13)
C22—C7—C16—C17	31.25 (15)	C24—C22—N3—C17	167.41 (13)
C18—C16—C17—N3	−132.70 (14)	C23—C22—N3—C17	−75.32 (16)
C7—C16—C17—N3	−7.14 (17)	C7—C22—N3—C17	42.19 (15)
C17—C16—C18—O2	−11.0 (3)	C16—C17—N3—C21	−150.84 (14)
C7—C16—C18—O2	−131.0 (2)	C16—C17—N3—C22	−22.53 (17)
C17—C16—C18—O3	169.66 (15)	O4—C23—N4—C29	−173.51 (17)
C7—C16—C18—O3	49.6 (2)	C22—C23—N4—C29	3.64 (19)
C5—C7—C22—N3	77.92 (14)	C28—C29—N4—C23	178.30 (18)
C8—C7—C22—N3	−168.10 (12)	C24—C29—N4—C23	0.5 (2)
C16—C7—C22—N3	−44.31 (13)	O2—C18—O3—C19	1.6 (3)
C5—C7—C22—C24	−48.38 (17)	C16—C18—O3—C19	−179.00 (19)
C8—C7—C22—C24	65.60 (16)	C20—C19—O3—C18	93.5 (4)
C16—C7—C22—C24	−170.61 (12)		

### Hydrogen-bond geometry (Å, º)

**Table:**

*D*—H···*A*	*D*—H	H···*A*	*D*···*A*	*D*—H···*A*
N4—H4···O4^i^	0.86	1.98	2.808 (2)	160
C20—H20*C*···O1^ii^	0.96	2.53	3.369 (6)	146

Symmetry codes: (i) −*x*+1, −*y*, −*z*; (ii) −*x*, −*y*, −*z*+1.

## References

[bb1] Bruker. (2008). *APEX2*, *SAINT* and *SADABS* Bruker AXS Inc., Madison, Wisconsin, U. S. A.

[bb2] Cremer, D. & Pople, J. A. (1975). *J. Am. Chem. Soc.* **97**, 1354-1358.

[bb3] Farrugia, L. J. (2012). *J. Appl. Cryst.* **45**, 849–854.

[bb4] Li, M.-J. & Feng, C.-J. (2009). *Acta Cryst.* E**65**, o2145. 10.1107/S1600536809031328 PMC2970036 21577554

[bb5] Li, D.-L., Wu, Y., Wang, Q., He, G. & Yu, L.-T. (2010). *Acta Cryst.* E**66**, o447. 10.1107/S1600536810002631 PMC2979868 21579862

[bb6] Pandeya, S. N., Sriram, D., Nath, G. & Declera, E. (1999). *Pharm. Acta Helv.* **74**, 11–17. 10.1016/s0031-6865(99)00010-2 10748620

[bb7] Priya, M. G. R., Srinivasan, T., Girija, K., Chandran, N. R. & Velmurugan, D. (2011*a*). *Acta Cryst.* E**67**, o2310. 10.1107/S1600536811030935 PMC3200588 22058942

[bb8] Priya, M. G. R., Zulykama, Y., Girija, K., Murugesh, S. & Perumal, P. T. (2011*b*). *Indian J. Chem. Sect.* B, **50**, 98–102.

[bb9] Sheldrick, G. M. (2008). *Acta Cryst.* A**64**, 112–122. 10.1107/S0108767307043930 18156677

[bb10] Spek, A. L. (2009). *Acta Cryst.* D**65**, 148–155. 10.1107/S090744490804362X PMC2631630 19171970

[bb11] Tereshima, K., Shimamura, H., Kawase, A., Tanaka, Y., Tanimura, T., Ishizuka, Y. & Sato, M. (1995). *Chem. Pharm. Bull.* **45**, 2021–2023. 10.1248/cpb.43.2021 8575039

[bb12] Wolfe, J. F., Rathman, T. L., Sleevi, M. C., Campbell, J. S. A. & Greenwood, T. D. (1990). *J. Med. Chem.* **33**, 161–166. 10.1021/jm00163a027 2296016

